# Isopropyl 3,4,5-trihy­droxy­benzoate

**DOI:** 10.1107/S1600536812004278

**Published:** 2012-02-10

**Authors:** Wei Lan, Xu-Ji Shen, Chao-Ni Xiao, Shi-Xiang Wang, Xiao-Hui Zheng

**Affiliations:** a College of Life Sciences, Northwest University, Xi’an 710069, People’s Republic of China; b Resource Biology and Biotechnology in Western China, Ministry of Education Key Laboratory of Northwest University, Xi’an 710069, People’s Republic of China

## Abstract

In the title compound, C_10_H_12_O_5_, the dihedral angle between the benzene ring is almost coplanar with the attached C(O)—O—C group [dihedral angle = 0.32 (15)°]. In the crystal, two intermolecular O—H⋯O hydrogen bonds make *R*_4_^4^(26) ring mofits.

## Related literature

For the properties of isopropyl gallate, see: Calheiros *et al.* (2008 ▶); Morais *et al.* (2010 ▶). For the synthesis method, see: Christiansen (1926 ▶); Li *et al.* (2001 ▶). For the hydrogen-bonding pattern, see: Bernstein *et al.* (1995 ▶).
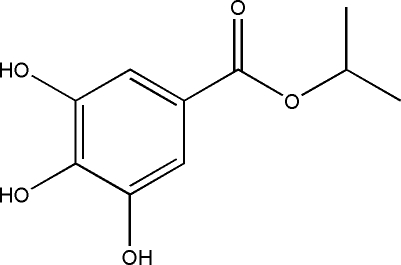


## Experimental

### 

#### Crystal data

C_10_H_12_O_5_
*M* *_r_* = 212.20
Monoclinic, 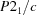

*a* = 19.148 (6) Å
*b* = 4.7030 (15) Å
*c* = 11.571 (4) Å
β = 90.159 (5)°
*V* = 1042.0 (6) Å^3^
*Z* = 4
Mo *K*α radiation
μ = 0.11 mm^−1^
*T* = 296 K
0.31 × 0.29 × 0.21 mm

#### Data collection

Bruker APEXII CCD diffractometer
Absorption correction: multi-scan (*SADABS*; Sheldrick, 1996 ▶) *T*_min_ = 0.967, *T*_max_ = 0.977
5181 measured reflections
2055 independent reflections
1589 reflections with *I* > 2σ(*I*)
*R*_int_ = 0.033

#### Refinement

*R*[*F* ^2^ > 2σ(*F* ^2^)] = 0.042
*wR*(*F* ^2^) = 0.132
*S* = 1.05
2055 reflections
141 parameters
H-atom parameters constrained
Δρ_max_ = 0.18 e Å^−3^
Δρ_min_ = −0.18 e Å^−3^

### 

Data collection: *APEX2* (Bruker, 2009 ▶); cell refinement: *SAINT* (Bruker, 2009 ▶); data reduction: *SAINT*; program(s) used to solve structure: *SHELXS97* (Sheldrick, 2008 ▶); program(s) used to refine structure: *SHELXL97* (Sheldrick, 2008 ▶); molecular graphics: *SHELXTL* (Sheldrick, 2008 ▶); software used to prepare material for publication: *SHELXTL*.

## Supplementary Material

Crystal structure: contains datablock(s) I, global. DOI: 10.1107/S1600536812004278/mw2052sup1.cif

Structure factors: contains datablock(s) I. DOI: 10.1107/S1600536812004278/mw2052Isup2.hkl

Supplementary material file. DOI: 10.1107/S1600536812004278/mw2052Isup3.cml

Additional supplementary materials:  crystallographic information; 3D view; checkCIF report

## Figures and Tables

**Table 1:** Hydrogen-bond geometry (Å, °)

*D*—H⋯*A*	*D*—H	H⋯*A*	*D*⋯*A*	*D*—H⋯*A*
O1—H1⋯O1^i^	0.82	2.00	2.772 (2)	158
O3—H3⋯O4^ii^	0.82	1.93	2.742 (2)	173

Symmetry codes: (i) 
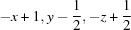
; (ii) 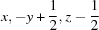.

**Fig. 1.**
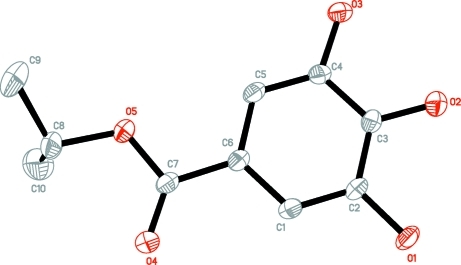
The molecular structure of (I) with the atom numbering scheme, showing displacement ellipsoids at the 30% probability level.

**Fig. 2.**
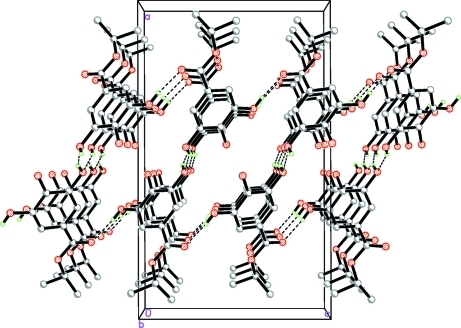
The packing of (I) viewed down the a axis with O—H···O hydrogen bonds shown as dashed lines.

## supplementary crystallographic information

### Comment

Pharmacological studies indicate the title compound, (I), has antioxidant, anti-apoptotic and anti-platelet activities suggesting it could be a new drug with therapeutic effects on cardiovascular or cerebrovascular diseases. (Calheiros *et al.*, 2008; Morais *et al.*, 2010).

The structure of the title compound, (I), is shown in Fig. 1. In the crystal, two intermolecular O—H···O hydrogen bonds make *R*_4_^4^(26) ring mofits (Bernstein *et al.*, 1995) which links the molecules into one-dimensional chains along [001] (Fig. 2).

### Experimental

0.01*M p*-toluenesulfonic acid in 2-propanol was added to a solution of 0.1*M* gallic acid in 500 ml of 2-propanol at room temperature. After being stirred and refluxed for 16 h, the solvent was removed under reduced pressure and the residue was extracted three times with ethyl acetate and filtered. The filtrate was washed successively with dilute saturated aqueous NaHCO_3_ solution, saturated aqueous NaCl solution, dried over MgSO_4_ and was evaporated to dryness. The crude product was purified by chromatography (SiO_2_; elution with petroleum ether and ethyl acetate, 5:1 *v*/*v*). Yield 36%. (Christiansen, 1926; Li *et al.*, 2001).

X-ray quality crystals were obtained from a solution of the title compound in acetone and toluene at room temperature. Spectroscopic analysis: IR (KBr, cm^-1^): 3499, 2971, 2922, 2957, 1677, 1609, 1671, 1613, 1541, 1449, 1327, 1252, 1165, 1111, 1026, 979; ^1^H NMR (DMSO, δ, p.p.m.): 9.126(s, 3 H), 6.946(s, 2 H), 5.014—5.055(m, 1 H), 1.274 (s, 3H), 1.264 (s, 3 H).

### Refinement

H atoms bonded to O atoms were located in a difference map and their positions adjusted to give O—H = 0.82 Å. Other H atoms were positioned geometrically with C—H = 0.93–0.96 Å. All were included as riding contributions (including free rotation about the ethanol C—C bond) with *U*_iso_(H) = 1.2*U*_eq_(O or C) or 1.5*U*_eq_(C).

### Crystal data

**Table:**

C_10_H_12_O_5_	*F*(000) = 448
*M**_r_* = 212.20	*D*_x_ = 1.353 Mg m^−^^3^
Monoclinic, *P*2_1_/*c*	Melting point: 396(1) K
Hall symbol: -P 2ybc	Mo *K*α radiation, λ = 0.71073 Å
*a* = 19.148 (6) Å	Cell parameters from 1697 reflections
*b* = 4.7030 (15) Å	θ = 3.5–25.7°
*c* = 11.571 (4) Å	µ = 0.11 mm^−^^1^
β = 90.159 (5)°	*T* = 296 K
*V* = 1042.0 (6) Å^3^	Block, colourless
*Z* = 4	0.31 × 0.29 × 0.21 mm

### Data collection

**Table:**

Bruker APEXII CCD diffractometer	2055 independent reflections
Radiation source: fine-focus sealed tube	1589 reflections with *I* > 2σ(*I*)
Graphite monochromator	*R*_int_ = 0.033
φ and ω scans	θ_max_ = 26.1°, θ_min_ = 3.5°
Absorption correction: multi-scan (*SADABS*; Sheldrick, 1996)	*h* = −21→23
*T*_min_ = 0.967, *T*_max_ = 0.977	*k* = −5→5
5181 measured reflections	*l* = −14→12

### Refinement

**Table:**

Refinement on *F*^2^	Primary atom site location: structure-invariant direct methods
Least-squares matrix: full	Secondary atom site location: difference Fourier map
*R*[*F*^2^ > 2σ(*F*^2^)] = 0.042	Hydrogen site location: inferred from neighbouring sites
*wR*(*F*^2^) = 0.132	H-atom parameters constrained
*S* = 1.05	*w* = 1/[σ^2^(*F*_o_^2^) + (0.0778*P*)^2^ + 0.0222*P*] where *P* = (*F*_o_^2^ + 2*F*_c_^2^)/3
2055 reflections	(Δ/σ)_max_ < 0.001
141 parameters	Δρ_max_ = 0.18 e Å^−^^3^
0 restraints	Δρ_min_ = −0.18 e Å^−^^3^

### Special details

**Table:**

Geometry. All e.s.d.'s (except the e.s.d. in the dihedral angle between two l.s. planes) are estimated using the full covariance matrix. The cell e.s.d.'s are taken into account individually in the estimation of e.s.d.'s in distances, angles and torsion angles; correlations between e.s.d.'s in cell parameters are only used when they are defined by crystal symmetry. An approximate (isotropic) treatment of cell e.s.d.'s is used for estimating e.s.d.'s involving l.s. planes.
Refinement. Refinement of *F*^2^ against ALL reflections. The weighted *R*-factor *wR* and goodness of fit *S* are based on *F*^2^, conventional *R*-factors *R* are based on *F*, with *F* set to zero for negative *F*^2^. The threshold expression of *F*^2^ > σ(*F*^2^) is used only for calculating *R*-factors(gt) *etc*. and is not relevant to the choice of reflections for refinement. *R*-factors based on *F*^2^ are statistically about twice as large as those based on *F*, and *R*- factors based on ALL data will be even larger.

### Fractional atomic coordinates and isotropic or equivalent isotropic displacement parameters (Å^2^)

**Table:**

	*x*	*y*	*z*	*U*_iso_*/*U*_eq_	
O1	0.46197 (5)	−0.0043 (2)	0.24204 (10)	0.0447 (3)	
H1	0.4823	−0.1535	0.2275	0.067*	
O2	0.45282 (6)	−0.3102 (3)	0.04068 (10)	0.0478 (3)	
H2	0.4449	−0.3814	−0.0227	0.072*	
O3	0.34242 (6)	−0.2621 (3)	−0.09898 (9)	0.0485 (4)	
H3	0.3094	−0.2187	−0.1407	0.073*	
O4	0.23999 (6)	0.5891 (3)	0.25056 (9)	0.0462 (4)	
O5	0.18797 (6)	0.4562 (3)	0.08655 (10)	0.0476 (4)	
C1	0.35320 (8)	0.2096 (3)	0.20035 (12)	0.0349 (4)	
H1A	0.3564	0.3109	0.2692	0.042*	
C2	0.40505 (7)	0.0233 (3)	0.17032 (13)	0.0337 (4)	
C3	0.40050 (7)	−0.1299 (3)	0.06846 (12)	0.0341 (4)	
C4	0.34216 (8)	−0.0952 (3)	−0.00283 (12)	0.0339 (4)	
C5	0.29047 (8)	0.0934 (3)	0.02583 (12)	0.0354 (4)	
H5	0.2522	0.1181	−0.0227	0.042*	
C6	0.29573 (7)	0.2479 (3)	0.12825 (12)	0.0329 (4)	
C7	0.24087 (8)	0.4472 (3)	0.16310 (13)	0.0356 (4)	
C8	0.13112 (9)	0.6509 (4)	0.10811 (16)	0.0549 (5)	
H8	0.1488	0.8212	0.1473	0.066*	
C9	0.10338 (12)	0.7293 (5)	−0.0096 (2)	0.0821 (8)	
H9A	0.1394	0.8223	−0.0530	0.123*	
H9B	0.0643	0.8552	−0.0012	0.123*	
H9C	0.0888	0.5603	−0.0495	0.123*	
C10	0.07847 (11)	0.5047 (6)	0.1831 (2)	0.0847 (8)	
H10A	0.0618	0.3367	0.1448	0.127*	
H10B	0.0400	0.6308	0.1974	0.127*	
H10C	0.0999	0.4529	0.2552	0.127*	

### Atomic displacement parameters (Å^2^)

**Table:**

	*U*^11^	*U*^22^	*U*^33^	*U*^12^	*U*^13^	*U*^23^
O1	0.0373 (6)	0.0438 (7)	0.0530 (7)	0.0058 (5)	−0.0220 (5)	−0.0060 (5)
O2	0.0415 (7)	0.0523 (8)	0.0495 (7)	0.0136 (6)	−0.0065 (5)	−0.0082 (6)
O3	0.0558 (7)	0.0553 (8)	0.0344 (6)	0.0150 (6)	−0.0117 (5)	−0.0108 (5)
O4	0.0429 (7)	0.0565 (8)	0.0391 (7)	0.0089 (5)	−0.0089 (5)	−0.0104 (5)
O5	0.0350 (6)	0.0611 (8)	0.0467 (7)	0.0134 (5)	−0.0145 (5)	−0.0123 (5)
C1	0.0370 (8)	0.0361 (9)	0.0315 (8)	−0.0020 (7)	−0.0077 (6)	−0.0006 (6)
C2	0.0293 (7)	0.0349 (9)	0.0369 (8)	−0.0032 (6)	−0.0097 (6)	0.0038 (6)
C3	0.0322 (8)	0.0332 (8)	0.0370 (8)	0.0027 (6)	−0.0023 (6)	0.0043 (6)
C4	0.0367 (8)	0.0376 (9)	0.0274 (7)	0.0000 (6)	−0.0028 (6)	0.0007 (6)
C5	0.0333 (8)	0.0428 (9)	0.0300 (8)	0.0003 (6)	−0.0087 (6)	0.0030 (6)
C6	0.0308 (7)	0.0370 (9)	0.0310 (8)	−0.0003 (6)	−0.0044 (6)	0.0033 (6)
C7	0.0331 (8)	0.0429 (9)	0.0308 (8)	−0.0013 (7)	−0.0069 (6)	0.0018 (7)
C8	0.0375 (9)	0.0619 (13)	0.0653 (12)	0.0159 (9)	−0.0152 (8)	−0.0146 (10)
C9	0.0632 (13)	0.0969 (19)	0.0860 (16)	0.0249 (13)	−0.0316 (11)	0.0047 (13)
C10	0.0523 (13)	0.120 (2)	0.0817 (16)	0.0100 (13)	0.0083 (11)	−0.0198 (14)

### Geometric parameters (Å, º)

**Table:**

O1—C2	1.3741 (16)	C4—C5	1.371 (2)
O1—H1	0.8200	C5—C6	1.394 (2)
O2—C3	1.3519 (18)	C5—H5	0.9300
O2—H2	0.8200	C6—C7	1.465 (2)
O3—C4	1.3616 (19)	C8—C10	1.499 (3)
O3—H3	0.8200	C8—C9	1.506 (3)
O4—C7	1.2122 (19)	C8—H8	0.9800
O5—C7	1.3443 (17)	C9—H9A	0.9600
O5—C8	1.445 (2)	C9—H9B	0.9600
C1—C2	1.370 (2)	C9—H9C	0.9600
C1—C6	1.3909 (19)	C10—H10A	0.9600
C1—H1A	0.9300	C10—H10B	0.9600
C2—C3	1.384 (2)	C10—H10C	0.9600
C3—C4	1.397 (2)		
			
C2—O1—H1	109.5	O4—C7—O5	121.38 (14)
C3—O2—H2	109.5	O4—C7—C6	126.39 (13)
C4—O3—H3	109.5	O5—C7—C6	112.22 (13)
C7—O5—C8	118.22 (13)	O5—C8—C10	108.52 (18)
C2—C1—C6	120.23 (14)	O5—C8—C9	105.25 (16)
C2—C1—H1A	119.9	C10—C8—C9	113.57 (18)
C6—C1—H1A	119.9	O5—C8—H8	109.8
C1—C2—O1	118.78 (13)	C10—C8—H8	109.8
C1—C2—C3	120.34 (13)	C9—C8—H8	109.8
O1—C2—C3	120.86 (14)	C8—C9—H9A	109.5
O2—C3—C2	118.96 (12)	C8—C9—H9B	109.5
O2—C3—C4	121.67 (13)	H9A—C9—H9B	109.5
C2—C3—C4	119.37 (14)	C8—C9—H9C	109.5
O3—C4—C5	125.10 (13)	H9A—C9—H9C	109.5
O3—C4—C3	114.24 (14)	H9B—C9—H9C	109.5
C5—C4—C3	120.66 (13)	C8—C10—H10A	109.5
C4—C5—C6	119.51 (13)	C8—C10—H10B	109.5
C4—C5—H5	120.2	H10A—C10—H10B	109.5
C6—C5—H5	120.2	C8—C10—H10C	109.5
C1—C6—C5	119.86 (14)	H10A—C10—H10C	109.5
C1—C6—C7	118.98 (13)	H10B—C10—H10C	109.5
C5—C6—C7	121.15 (13)		
			
C6—C1—C2—O1	178.39 (14)	C2—C1—C6—C5	0.8 (2)
C6—C1—C2—C3	−0.5 (2)	C2—C1—C6—C7	179.57 (13)
C1—C2—C3—O2	179.39 (13)	C4—C5—C6—C1	0.0 (2)
O1—C2—C3—O2	0.6 (2)	C4—C5—C6—C7	−178.73 (13)
C1—C2—C3—C4	−0.7 (2)	C8—O5—C7—O4	3.0 (2)
O1—C2—C3—C4	−179.53 (13)	C8—O5—C7—C6	−178.08 (14)
O2—C3—C4—O3	1.6 (2)	C1—C6—C7—O4	0.5 (2)
C2—C3—C4—O3	−178.31 (14)	C5—C6—C7—O4	179.19 (16)
O2—C3—C4—C5	−178.57 (14)	C1—C6—C7—O5	−178.35 (13)
C2—C3—C4—C5	1.5 (2)	C5—C6—C7—O5	0.4 (2)
O3—C4—C5—C6	178.66 (14)	C7—O5—C8—C10	−87.29 (19)
C3—C4—C5—C6	−1.2 (2)	C7—O5—C8—C9	150.82 (17)

### Hydrogen-bond geometry (Å, º)

**Table:**

*D*—H···*A*	*D*—H	H···*A*	*D*···*A*	*D*—H···*A*
O1—H1···O1^i^	0.82	2.00	2.772 (2)	158
O3—H3···O4^ii^	0.82	1.93	2.742 (2)	173

Symmetry codes: (i) −*x*+1, *y*−1/2, −*z*+1/2; (ii) *x*, −*y*+1/2, *z*−1/2.
